# Prosthetic Neck Fracture of a Cementless Titanium Femoral Stem

**DOI:** 10.7759/cureus.71508

**Published:** 2024-10-15

**Authors:** Zakhia Michael Malhame, Fouad Lamnaouar, Abdel Massih Abou Chaaya

**Affiliations:** 1 Orthopedics and Traumatology, Centre Hospitalier Victor Dupouy, Argenteuil, FRA; 2 Orthopedics and Traumatology, Holy Spirit University of Kaslik, Byblos, LBN

**Keywords:** cementless total hip arthroplasty, femorotomy, prosthetic neck fracture, stress-fracture, titanium implant

## Abstract

This case report presents a unique instance of a prosthetic neck fracture in an 81-year-old male with a CERAVER CERAFIT titanium (Ti6Al4V) femoral implant paired with a ceramic-on-ceramic total hip replacement. No fractures of the neck of a titanium femoral stem coated with hydroxy-apatite have been reported in the literature for this type of implant. The patient experienced a sudden, non-traumatic fracture, many years after his primary surgery. The report discusses the clinical presentation, diagnostic findings, known risk factors for femoral neck implant fractures, surgical management, and the outcome.

## Introduction

Total hip arthroplasty (THA) is a widely performed procedure, and it can significantly improve the quality of life in patients with debilitating hip conditions such as osteoarthritis [1]. The success of THA largely depends on the durability of the implant materials used. Modern hip prostheses typically comprise various alloys, including titanium alloys, cobalt-chromium alloys, and stainless steel [2]. Each of these materials has its advantages in terms of biocompatibility, strength, and fatigue resistance, but they are not immune to complications such as fractures [3].

Femoral neck fractures, associated with hip implants, are rare but can occur due to several factors, such as the mechanical properties of the alloy, the duration of implantation, the patient’s activity level, and changes in bone quality over time [3]. Fatigue fractures may develop due to cyclic loading and repeated stress on the implant, especially in patients who remain physically active or those who have implants for extended periods [4]. Prosthetic neck fractures can be broadly categorized into head-neck fractures and neck-shoulder fractures [3]. This complication presents significant challenges in orthopedic management and may necessitate complex revision surgeries. This report discusses the case of a femoral neck fracture in a patient with a long-standing titanium hip implant, highlighting the risk factors, surgical management, and outcome.

## Case presentation

Patient history

The patient was an 81-year-old male with a history of type 2 diabetes mellitus and hypertension, both of which are well managed with medication. His surgical history included a partial meniscectomy of the left knee in 1987 and a bilateral total knee replacement (TKR) seven years ago. He had a history of right hip osteoarthritis and had undergone THA 24 years before the incident, using a posterolateral approach to the hip. The implant was a non-cemented CERAVER CERAFIT femoral implant size 10 and an acetabular implant size 52 with a head size of 32 long neck. The chosen friction couple was ceramic-ceramic. Notably, He had returned only once to see his surgeon, two months postoperatively for routine evaluation with a good outcome and complete recovery. He had been lost to follow-up after this last appointment. He was a regular tennis player for 17 years following the surgery and occasionally played soccer. He presented to the emergency department with sudden-onset severe pain in his right hip while standing at home. No traumatic event, such as a fall or accident, was reported. He was unable to bear weight on the affected limb. Physical examination revealed significant tenderness over the right hip, limited range of motion, shortening of the lower limb, and an external rotation of the hip.

Initial workup

**Figure 1:**
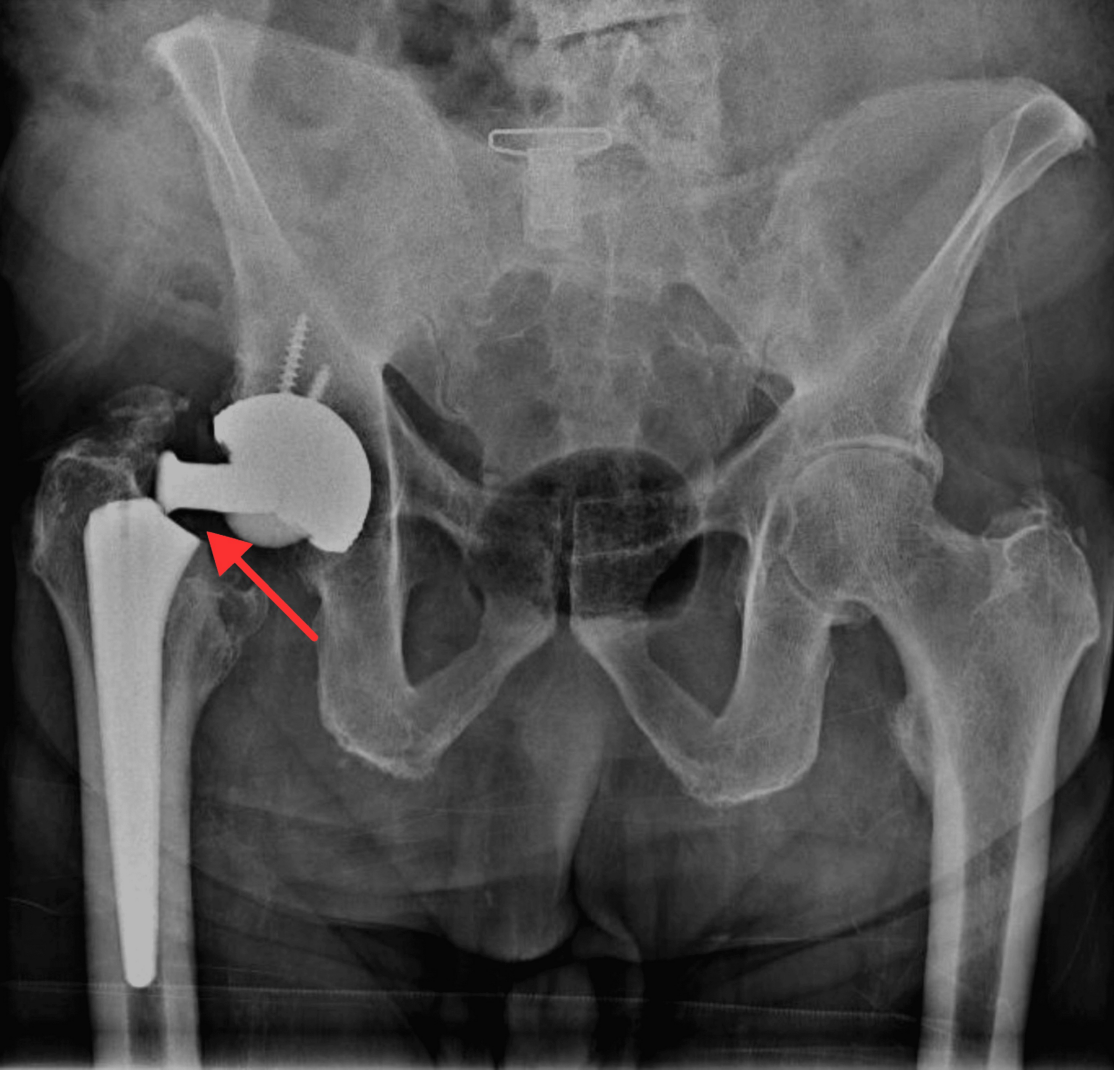
Anteroposterior view of the pelvis showing the prosthetic neck fracture at the time of the failure Red arrow shows the prosthetic fracture at the neck-shoulder level

Initial radiographs revealed a displaced femoral prosthetic neck fracture at the neck-shoulder junction of the femoral stem. The femoral stem and the acetabular component appeared intact, with no signs of loosening or dislocation (Figure 1).

Biology test results on admission showed a C-reactive protein (CRP) of 1 mg/L (normal value: <5 mg/L), a creatinine level of 154 µmol/L with 9000 leucocytes and a hemoglobin of 14 g/dL. Vitamin D levels were normal (he was on regular supplementation with cholecalciferol (vitamin D3) (Table 1).

**Table 1:** Blood workup WBCs: white blood cells

Parameters	Patient values	Normal ranges
C-reactive protein, mg/L	1	<5
Creatinine, µmol/L	154	65–120
Leucocytes, WBCs/μL	9000	4,500–11,000
Hemoglobin, g/dL	14	3.8–17.2
Vitamin D, nmol/L	112	50–125

Management

**Figure 2:**
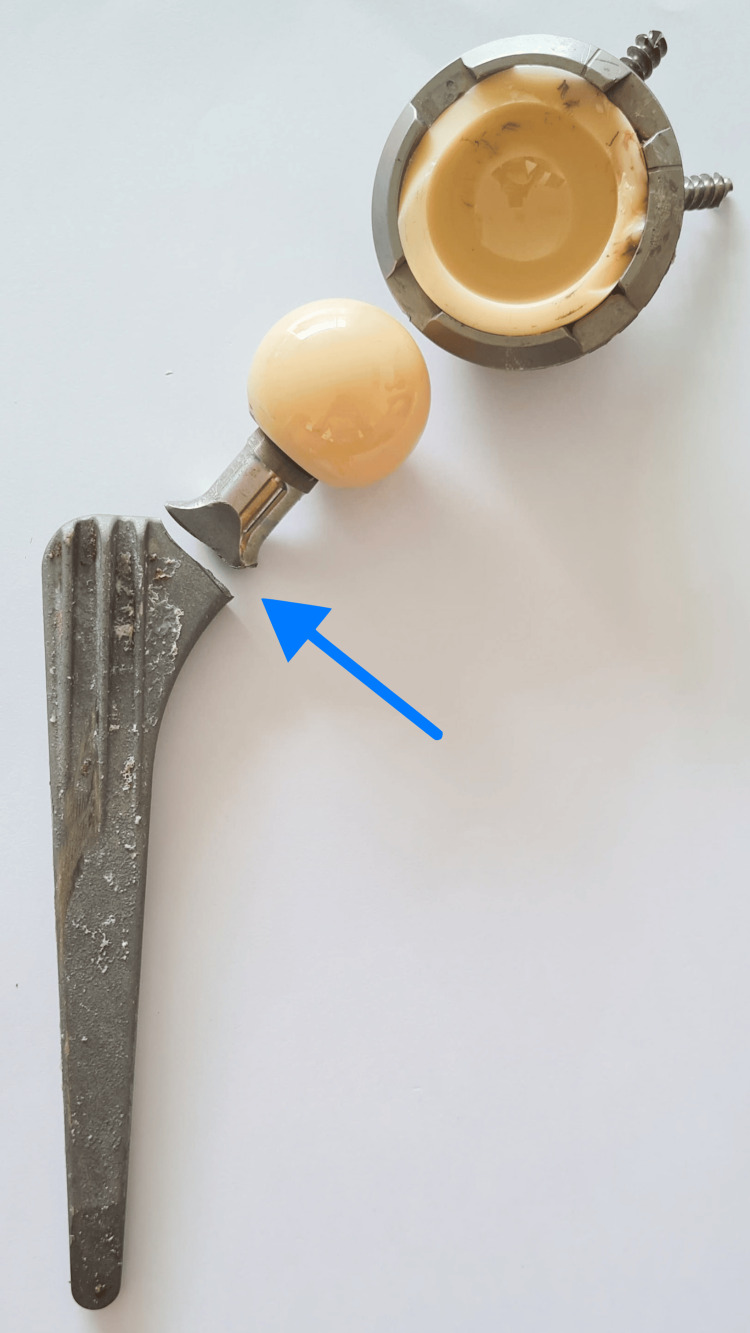
Postoperative picture of the broken femoral stem The neck is broken with a transverse pattern at the neck-shoulder junction. The blue arrow shows the transverse fracture pattern at the neck-shoulder junction of the femoral stem

Given the severity of the fracture and the advanced age of the implant, the orthopedic team decided to proceed with total hip revision surgery. The original stem, which was made of titanium alloy (Ti6Al4V) and not cemented, added complexity to the surgical approach. The surgery was performed using a trochanteric approach to the hip, making an incision of approximately 20 cm in length, and involved performing a cortical window femoral osteotomy of the trochantero-diaphyseal region of 12 cm in length to gain access to the femoral implant. The fracture was found to involve the neck-shoulder region as predicted in the preoperative radiographs (Figure 2).

There was no sign of impingement, and no metal debris was found in the periarticular soft tissues. Despite the osteotomy, the implant had an excellent fixation but was loosened progressively and removed using a chisel with minimal bone damage. Preparation of the medullary canal was done with reamers. A collared revision stem with hydroxyapatite coating (EVOLUTIS STEMSYS) size 12L was placed with very good primary fixation. The trochanterotomy was fixed with a trochanteric hook (STRYKER) with two metallic cables. The femorotomy window was closed with two Dall-Miles cables on the distal diaphyseal part, resulting in a stable synthesis.

On the acetabular side, the ceramic insert was blocked inside the acetabular cup and impossible to remove, preventing access to the screws. The decision was made to remove the acetabular component in one block using a graft pusher. The acetabular cup was easily loosened and taken out without any damage to the bone. The bony acetabulum maintained a spherical continuity, and, after reaming, a definitive dual-mobility acetabular cup (X-NOV), size 50, was placed with an excellent primary fixation.

**Figure 3:**
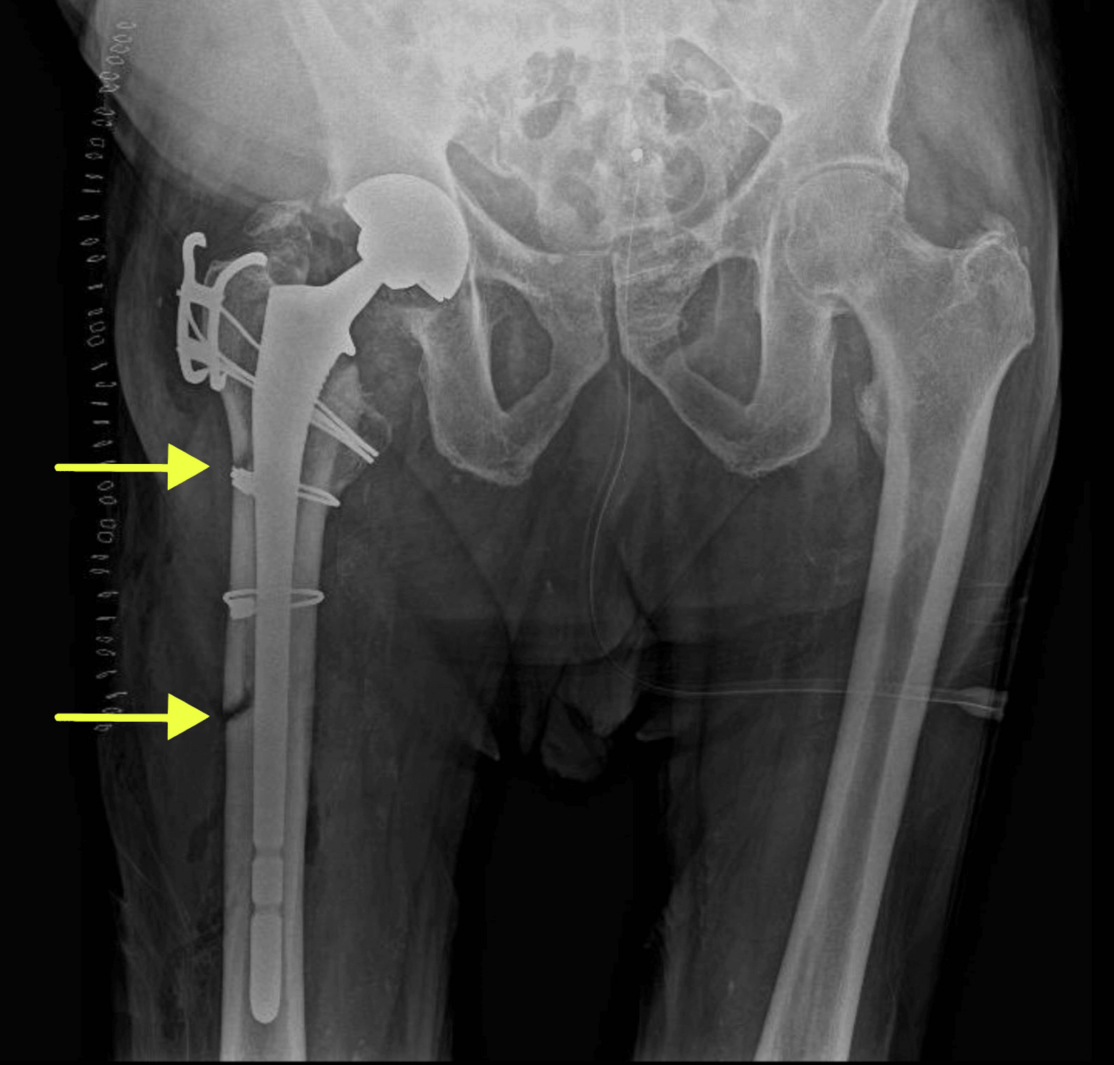
Anteroposterior view of the pelvis directly immediately revision surgery Yellow arrows show the site of the femorotomy

A metallic head, diameter 28, medium neck, and a dual mobility polyethylene insert 50/28 were chosen. The prosthesis was stable and mobile with intraoperative testing. Postoperative radiographs confirmed satisfactory alignment and stable fixation of the new implants (Figure 3).

Outcome and follow-up

**Figure 4:**
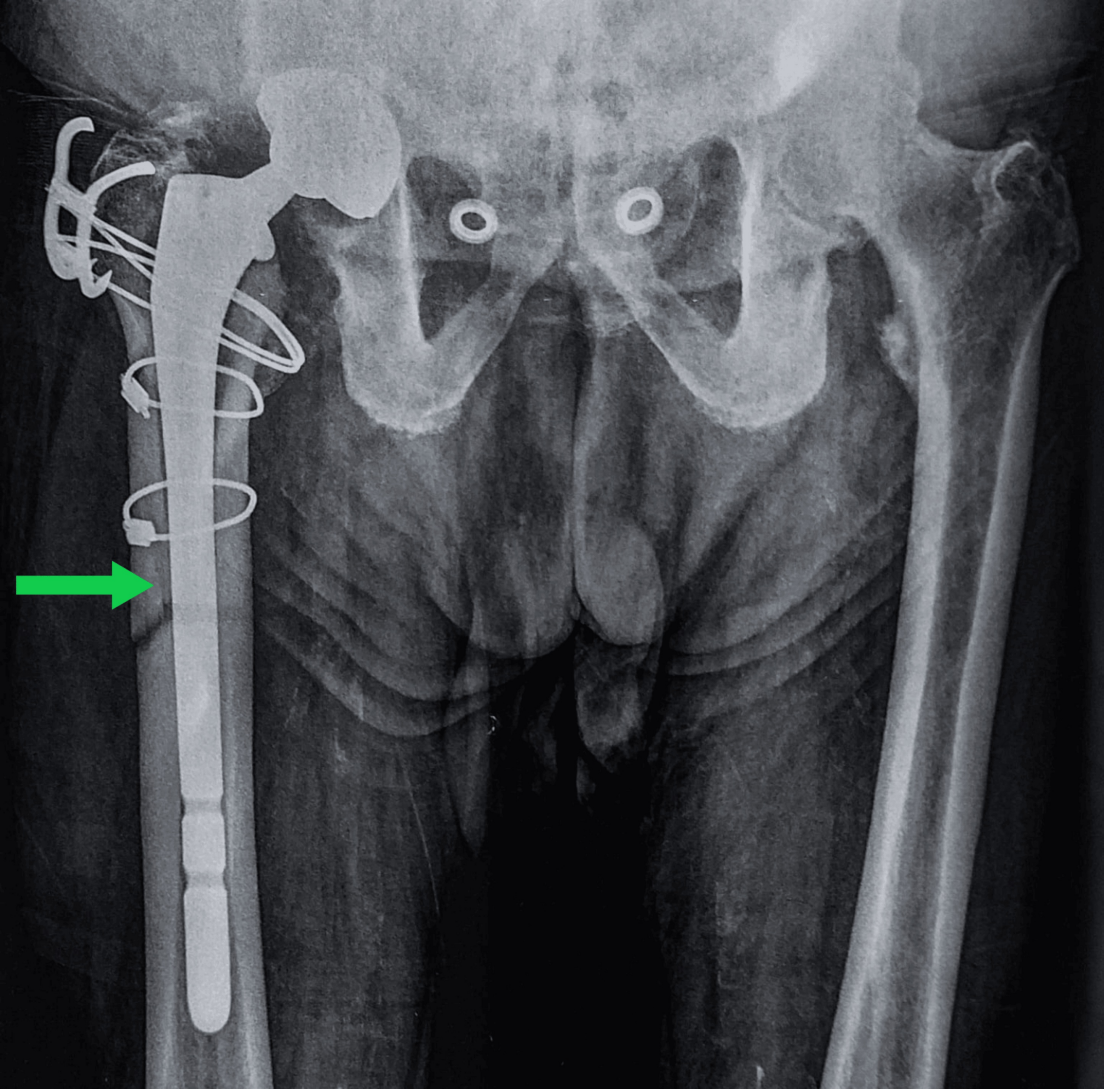
Anteroposterior view of the pelvis two months after revision surgery The green arrow shows an area of integration of the new prosthetic components

The patient’s postoperative recovery was uneventful, and he was initially mobilized with partial weight-bearing for six weeks. At the two-month follow-up, he reported minimal pain and had regained near-normal function in the affected limb. Follow-up radiographs demonstrated satisfying integration of the new prosthetic components (Figure 4).

## Discussion

The rate of femoral component fracture is rare: around 0.27% as reported by Heck et al. in a retrospective survey conducted by the American Association of Hip and Knee Surgeons in 1995, including more than 60,000 hip arthroplasties [5]. Femoral neck fractures in patients with titanium hip implants, particularly on non-modular implants such as the CERAVER CERAFIT, are even rarer in the literature and present unique management challenges. Specific studies on the fracture of the femoral stem’s neck of a CERAVER CERAFIT were not found in the literature, but the general findings on titanium stem failures are relevant for understanding potential risks. The literature reveals that while titanium stems, including those made from Ti6Al4V, are generally durable, they can still be susceptible to fatigue fractures under certain conditions, particularly in long-term use or high-activity patients [3-6].

Non-modular implants have a fixed design and are intended to provide stability and strength. This can be advantageous in terms of reducing potential points of failure and simplifying the surgical procedure, though it may also limit flexibility compared to modular systems [6-7]. Hip neck fractures can be broadly categorized into two types; head-neck fractures and shoulder-neck fractures [3]. The first category occurs at the junction between the femoral head and neck. They are often associated with high cyclic loading and can be influenced by implant positioning and the stresses distributed through the implant. As for the neck-shoulder fractures, they occur at the transition between the femoral neck and the prosthetic shoulder, often where the implant stem meets the bone, as in our patient. These fractures can be influenced by the mechanical properties of the implant, such as the titanium alloy used, and the quality of the surrounding bone [3,7,8].

Several known risk factors can contribute to this type of fracture. Many studies have shown that male sex, young age, and high BMI were significant risk factors for femoral implant fracture [3-8]. The BMI of our patient was found to be 27.1 kg/m^2^, which falls under the overweight category, which could have contributed to increasing the risk of femoral implant fracture. The long duration of implantation is also a major risk factor [3-8]. Van Doesburg et al. showed that the prosthetic neck fracture appeared after seven years of THA on average [3]. Turnbull et al. demonstrated that 83% of stem fractures occurred before 10 years, with a minority occurring beyond 15 years [8]. Our patient’s implant had been in place for 24 years, making this case more unique, since prosthetic fracture is usually more of a medium rather than short- or long-term complication [9]. Another risk factor for prosthetic neck fracture is the patient’s high activity levels [8].

While ceramic and titanium alloy components are durable, prolonged mechanical stress over decades can increase the risk of fatigue fractures [1,3,7]. Despite his advanced age, our patient remained highly physically active for 17 years, regularly playing tennis and occasionally soccer. High-impact or repetitive activities can contribute to the wear and eventual fracture of the titanium stem [7]. The neck region is commonly the site of failure, as it experiences significant bending and torsional forces even during normal activities such as walking or running [3]. Another risk factor described in the literature, but was not applicable in our patient’s case, is the positioning of the implant in a varus alignment, increasing the mechanical stress on the stem and thereby increasing the risk of prosthetic fracture [8-10].

The management of this case involved a femorotomy window to remove the old implant and replace it with new components, which was deemed necessary due to the implant's non-cemented nature and its strong integration with the bone. [11]. The successful outcome in our patient demonstrates the effectiveness of a tailored surgical approach in managing such fractures associated with hip implants.

## Conclusions

This report highlights a rare but significant complication of a prosthetic femoral neck fracture in a patient with a CERAVER CERAFIT titanium femoral implant, occurring decades after the initial surgery and without a traumatic event. The management involved femorotomy and complete stem and cup replacement, leading to a successful outcome. Known risk factors, such as prolonged implantation, high activity levels, and aging bone quality, likely contributed to the fracture. The patient’s lack of follow-up care after the initial surgery may have contributed to an unmonitored decline in bone quality or implant integrity, underscoring the importance of regular postoperative evaluations. This report emphasizes the need for continuous monitoring of patients with long-standing implants, particularly those who remain physically active.
